# Fully Fabric-Based Triboelectric Nanogenerators as Self-Powered Human–Machine Interactive Keyboards

**DOI:** 10.1007/s40820-021-00621-7

**Published:** 2021-04-05

**Authors:** Jia Yi, Kai Dong, Shen Shen, Yang Jiang, Xiao Peng, Cuiying Ye, Zhong Lin Wang

**Affiliations:** 1 School of Physical Science and Technology, Guangxi University, Nanning, 530004 People’s Republic of China; 2 CAS Center for Excellence in Nanoscience, Beijing Key Laboratory of Micro-Nano Energy and Sensor, Beijing Institute of Nanoenergy and Nanosystems, Chinese Academy of Sciences, Beijing, 100083 People’s Republic of China; 3 School of Nanoscience and Technology, University of Chinese Academy of Sciences, Beijing, 100049 People’s Republic of China; 4 School of Material Science and Engineering, Georgia Institute of Technology, Atlanta, GA 30332-0245 USA

**Keywords:** Triboelectric nanogenerators, Self-powered keyboard, Human–machine interface, Wearable electronics, Fully fabric-based

## Abstract

Combination flexible and stretchable textiles with self-powered sensors bring a novel insight into wearable functional electronics and cyber security in the era of Internet of Things. This work presents a highly flexible and self-powered fully fabric-based triboelectric nanogenerator (F-TENG) with sandwiched structure for biomechanical energy harvesting and real-time biometric authentication. The prepared F-TENG can power a digital watch by low-frequency motion and respond to the pressure change by the fall of leaves. A self-powered wearable keyboard (SPWK) is also fabricated by integrating large-area F-TENG sensor arrays, which not only can trace and record electrophysiological signals, but also can identify individuals' typing characteristics by means of the Haar wavelet. Based on these merits, the SPWK has promising applications in the realm of wearable electronics, self-powered sensors, cyber security, and artificial intelligences.

**Supplementary Information:**

The online version contains supplementary material available at 10.1007/s40820-021-00621-7.

## Highlights

- A fully fabric-based mechanical energy harvester with a sandwich structure is developed, which can respond to the pressure change by the fall of leaves.
- A self-powered keyboard with the ability of biometric recognition is demonstrated, which is able to resist illegal intrusion by judging the keystroke behaviors.

**Supplementary Information:**

The online version contains supplementary material available at 10.1007/s40820-021-00621-7.

## Graphical abstract

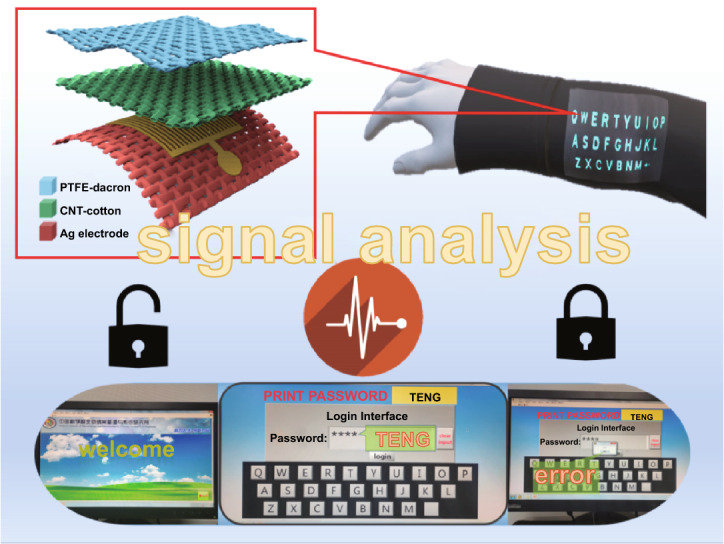

## Introduction

It is following a general trend of functionality, portability and self-security with the massive development of the fifth-generation electronic technology, especially when it comes to wearable electronics. A superb feature of this trend is the tremendous demand in electronic devices/systems, in a total of billions to trillions, each of which needs a mobile power source. The traditional mobile power sources have some defects, such as bulky volume, overbalance weight, and limited lifetime [1–4], which hinder the practical and sustainable applications of wearable electronics. Fortunately, since the invention of the triboelectric nanogenerator (TENG) in 2012 [5–7], wearable electronics provide new dawn and reengineering for sensing and energy harvesting [8–10]. On account of the advantages of TENGs, such as easy structural design [11, 12], low cost [13], high conversion efficiency [14, 15], and broad range of applications [16], the coupling effect of contact electrification (CE) and electrostatic induction can be achieved between any materials [6, 17]. Therefore, the integration of general-purpose TENG technology with smart fabrics brings new vitality and more possibilities to the next generation of wearable electronics [18, 19], personal healthcare [20, 21], and human–computer interfaces [22–24].

With the development of flexible human–machine interface devices, intensive progress has been realized by utilizing deformable conductors [25, 26], stretchable sensors [27, 28], or flexible films [29–31]. However, polymer substrates, such as polyethylene terephthalate (PET), polyimide (PI), and polydimethylsiloxane (PDMS), were used for most flexible human–machine interface devices [32, 33], have the disadvantage of low fit and unsatisfying comfort for human body. Textiles, due to their merits of hygroscopic, soft, breathable, and comfortable to human, are considered as ideal vehicles to design flexible human–machine interfacing devices, especially for wearable electronics with personal healthcare/biomedical monitoring or biometrics [10, 34–36]. Recently, self-powered smart textiles combined with TENGs for accommodating the era of IoTs have been reported [37–40]. For example, a 3D honeycomb structure woven fabric triboelectric nanogenerator based on flame-retardant wrapping yarn was developed for fire escape and rescue [11]. A DC fabric TENG, which takes advantage of electrostatic breakdown phenomenon of clothes, can light up 416 serially connected light-emitting diodes [7, 9]. In addition, the stretched yarns were embedded into TENGs can be used for motion monitoring [34, 41]. However, there are rarely reports on devices that integrate TENGs and sensors based on fully textiles, most of them were just assembled into the shape of fabric, which was lack of air permeability and comfortability. On the other hand, some intelligent human–computer interface devices have been developed based on fully fabrics, but their too simple functions to meet the needs of people's daily life and production. Thus, a device, composed of fully textile-based TENG, is required to implement the merits of comfortability, wearability, human–computer interaction, and multifunctional biosensing.

Here, a fully fabric-based pressure sensor array was manufactured via a simple and effective route, which was further assembled into a biometric SPWK to effectively prevent unauthorized computer access. The developed F-TENG prototype has appropriate stretchability and stability as well as high-pressure sensitivity, and can generate electrical signals to detect external keystroke. A trigger voltage threshold of 2 V was designed for the wearable keyboard system, so that it could work stably even in a severe noise environment. Furthermore, the Haar wavelet was employed to successfully classify and identify the typing signal. More importantly, on the basis of the electrical output signal obtained from different users, a biometric authentication system could access to the system by biometrics recognition not just the digital passwords were established. Upon the user logs into the system, the self-security function through matching specific users’ electrical signals was also verified.

Owning to the unique woven structure and advanced self-powered function, the SPWK has the characteristics of self-powered ability, self-assignment-safe and wearability. And it is portable due to the combination SPWK with fabrics, achieving "zero space" occupation. The SPWK is an intelligent device to convert mechanical energy into electrical energy through typing movement. Due to the high-pressure sensitivity of the sensor, it can dynamically identify and prevent unauthorized access. And it can make significant progress in biometric systems, which has versatile applications in wearable electronic, artificial intelligence, cyber security, and human–machine interaction.

## Experimental Section

### Fabrication of F-TENG

Fabrication of Ag conducting layer: The polymer fiber was polished into NaOH solution and rinsed with deionized water to remove impurities [42]. The processed polyester cloth was plated with a layer of Ag electrode by screen printing method. Finally, the polyester fabric was dried at room temperature.

Fabrication of CNT coating: The SDBS was dissolved in the deionized water, and the concentration of the solution was fixed to 10 wt%. At the same time, CNT/SDBS dispersion liquid with different contents is prepared by adding CNTs to them. By using the layer-by-layer self-assembly approach, a cotton cloth was put in the CNT/SDBS dispersion liquid, then removed and dried for 8 times.

Fabrication of Frictional layer: The polyester fabric, which was cleaned with deionized water, was put into PTFE solution for 3 min and then placed in a vacuum drying oven at 70 °C to dry for 5 min. After repeating the above steps three times, the samples were dried at 150 °C for two hours.

### Characterizations

The surface morphology was observed by SEM, and the electrical output performance was measured by the 6,514 electrometer and the linear motor. The NI USB-6218 was used to be convert electrical signals into digital signals.

## Results and Discussion

### Fundamental Measurement of F-TENG

**Fig. 1:**
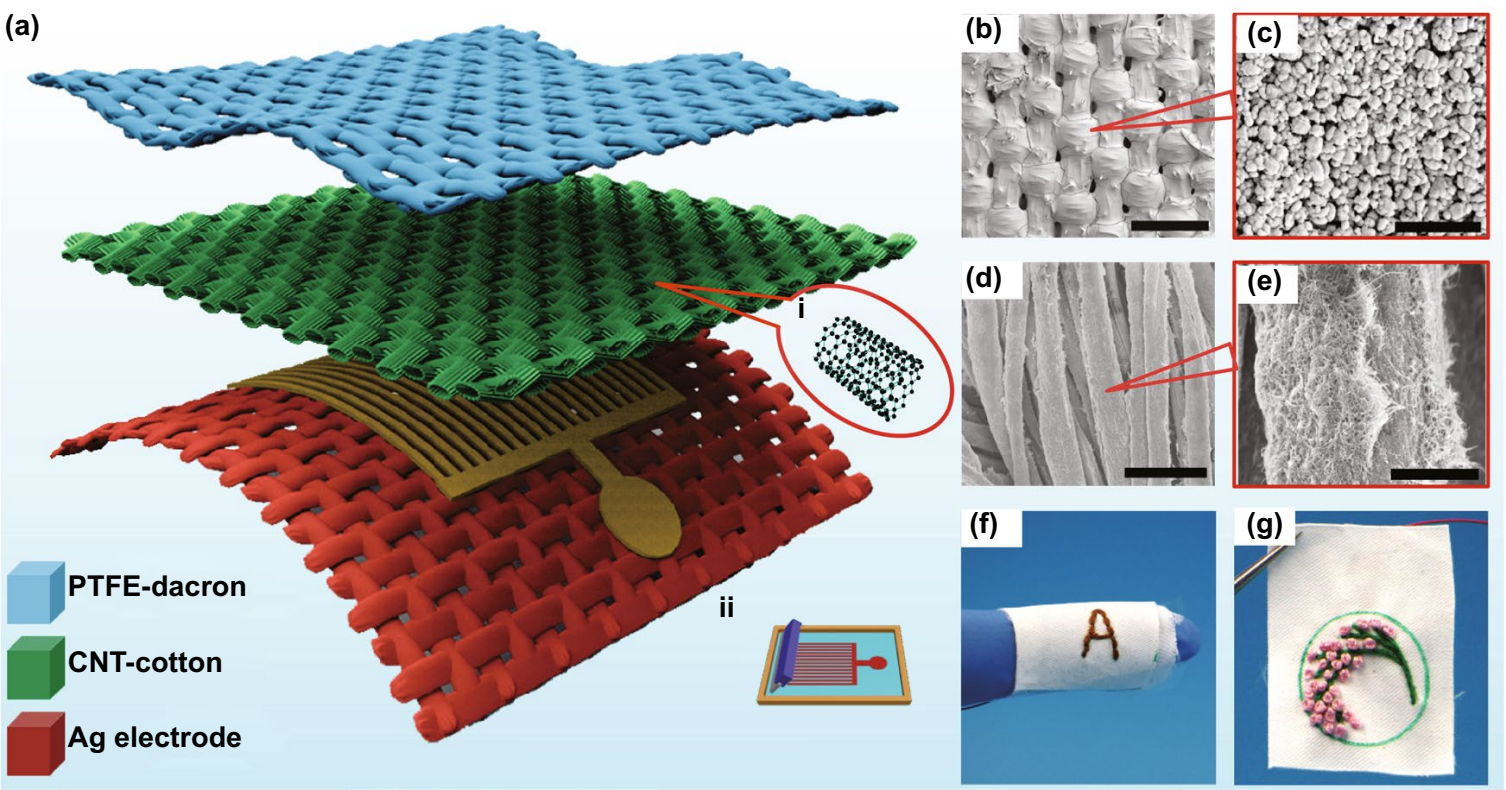
Structural design of the F-TENG. **a** Schematic diagram of the F-TENG. The insets **i** and **ii** show the molecular structure diagram and screen-printing process diagram of CNTs, respectively. **b-e** SEM images of the morphology of PTFE and CNT coated on the fabric at different magnification. The *scale bars* are 300 µm for (**b**), 1 µm for (**c**), 20 µm for (**d**), and 5 µm for (**e**). **f**, **g** Photographs showing that the F-TENG is under finger bending (**f**), or the form of embroidery (**g**)

The overall schematic of the developed F-TENG is shown in Fig. 1a. Firstly, the prepared polyester cloth which acts as friction layer was immersed into 60% polytetrafluoroethylene (PTFE) solution to obtain a uniformly mixed PTFE/polyester fabric. According to the SEM images of the polyester fabric (Fig. 1b, c) soaked in the PTFE solution, it can be seen that the PTFE particles are evenly distributed on the surface of the polyester fabric, the original air permeability and mechanical flexibility are well retained. In addition, carbon nanotubes (CNTs) were used to mix with the dispersant of sodium dodecylbenzene sulfonate (SDBS) to prepare CNT solution. A CNT/cotton conductive fabric was manufactured via a layer-by-layer self-assembly method until the resistivity was lower than 20 Ω cm (Fig. S1). The images in Fig. 1d, e show that CNTs were well integrated with the fabric, and each cotton fibers were decorated with a permeable CNT grid. Figure 1c, e is the partial enlarged views of Fig 1b, d, respectively. The silver electrode was pasted on the polyester fabric via screen printing in order to control the coated amount of silver slurry and the electrical conductivity of fabric electrode. The size of the interdigitated textile and the elemental mapping of the fabric surface are shown in Figs. S2 and S3, respectively. By designing different patterns on the surface of fabrics, the roughness can be drastically increased, which is a good approach to increasing the electrical performance. Figure 1f, g is the optical images of F-TENG after embroidering.

The gradient porous structure of CNT@Ag fabric provides F-TENG with a large surface area, sufficient roughness, and elasticity, leading to the variation of contact resistance under the pressure. Once the porous structures are compressed by external pressure, the surface of top CNT fabric will close contact the other bottom Ag electrode to create a rich conductive path (Fig. S4a). The LED lamp can emit light normally by connecting CNT/cotton fabric with the LED lamp under 3 V external power supply. The result further verified that CNTs are well coated on the cotton fibers, endowing the F-TENG an outstanding electrical conductivity (Fig. S4b).

**Fig. 2:**
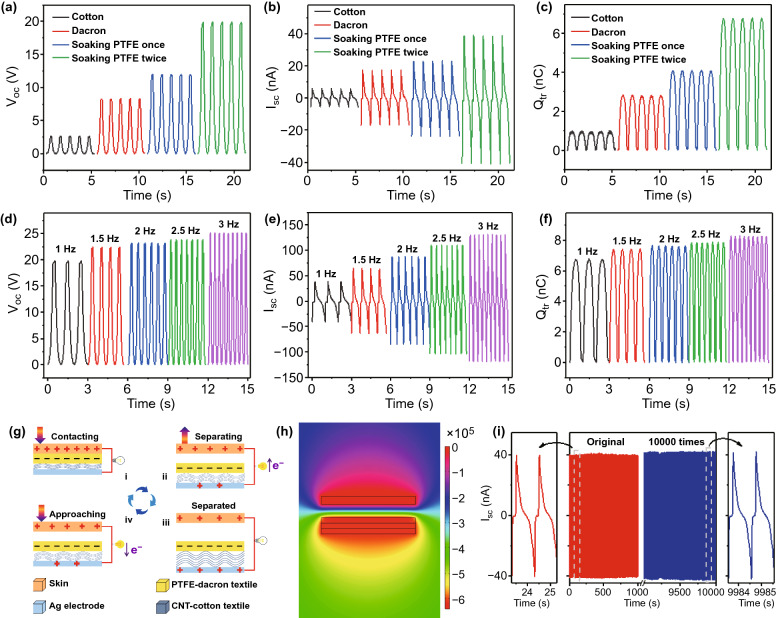
Electrical output performance and working mechanism of the F-TENG. **a–c** Effect of friction layers on the electrical output performance of the F-TENG, including **a** *V*_oc_, **b** *I*_sc_, and **c** *Q*_tr_. **d-f** Effect of the loading frequencies (1–3 Hz) on the electrical output performance of the F-TENG, including **d** *V*_oc_, **e** *I*_sc_ and **f** *Q*_tr_. **g** Schematic of the operation mechanism of the F-TENG in a single-electrode mode. **h** Simulation of the potential distribution of the F-TENG by using COMSOL software. **i** Long-term stability and durability test of the F-TENG under a pressure of 10 N

It is well known that different frictional layers can affect the electrical performance of TENGs [43, 44]. In order to investigate the effect of contact materials on the electrical output performance of the F-TENG, including open-circuit voltage (*V*_oc_), short-circuit current (*I*_sc_), and short-circuit charge transfer (*Q*_tr_), cotton fabric, dacron fabric, the fabric soaking with PTFE once, and the fabric soaking with PTFE twice are used as the contact materials, respectively. As the electrical output performance shown in Fig. 2a–c, the *V*_oc_ and *I*_sc_ reach maximum when the polyester fabric was treated by PTFE solution twice. The mechanical flexibility obviously declines with the increase in PTFE content, which is due to that the excessive PTFE nanoparticle can cover the fabric surface and form a layer of airtight film. Therefore, the proper content of the PTFE is critical for improving the output performance and permeability of F-TENG.

The frequency response of F-TENG with the optimal structural parameters is further analyzed. As shown in Fig. 2d, the Voc almost maintains its original state in spite of the variation of frequency, indicating that the F-TENG transfers equal triboelectric charges on the friction layer and generates the same static charges on both ends of the electrode, hence maintaining the equal potential difference between the two electrodes. According to the formula,

1 $$I = N \times e \times s \times v$$

where *N* is the number of transferred electrons, *e* is the charge of electron, *s* is the cross-sectional area of electron transport, and *v* is the electron transport rate. As the frequency increases, the electron transfer rate *v* increases, and eventually leads to an increase in *I*sc from 40 to 140 nA (Fig. 2e). After 1000 cycles of loading and unloading, the *I*_sc_ remains at 40 nA with no obvious deterioration (Fig. 2i), confirming that the electrical output of F-TENG possesses robust stability and excellent practical characteristics.

Figure 2g is the schematic diagram of the F-TENG in the contact and separation mode. In the initial state, the F-TENG contacts with human skin, triboelectrification effect occurs at the interface and generates the same number of charges with opposite polarities at the surface of skin and fabric layers, respectively (Fig. 2g-i). At this moment, considering that the two friction layers almost overlap, there is no practical electrical potential difference between the two surfaces. Once the two surfaces are gradually separated, positive charges on the conductive layer of CNT-cotton would be induced due to the electrostatic induction effect, allowing free electrons flow through Ag electrode to the ground (Fig. 2 g-ii). In the case of completed separation, there is no electrical signal on the surface of the tribological layer, corresponding to the neutralization of positive and negative charges during this period (Fig. 2 g-iii). It is important that the accumulated charges do not completely annihilate. On the contrary, they would remain for a long period of time due to the inherent characteristics of electronic insulation of friction layer. If the skin approaches the top surface of F-TENG, the positive charges on the CNT-cotton layer are induced, resulting in the opposite electrons flow from ground to the CNT-cotton layer to compensate for the potential difference (Fig. 2 g-iv). After the entire system returns to the original state (Fig. 2g-i), the friction layer completely offsets the positive and negative charge on the human skin. Thus, the contact and separation between human skin and F-TENG will generate instantaneous alternative current (AC) potential and current through external resistance. In order to well understand the power generation process, the theoretical model of F-TENG was established. And a simple finite element simulation was also conducted through COMSOL Multiphysics to observe the potential distribution in a completely separated state (Fig. 2h).

**Fig. 3:**
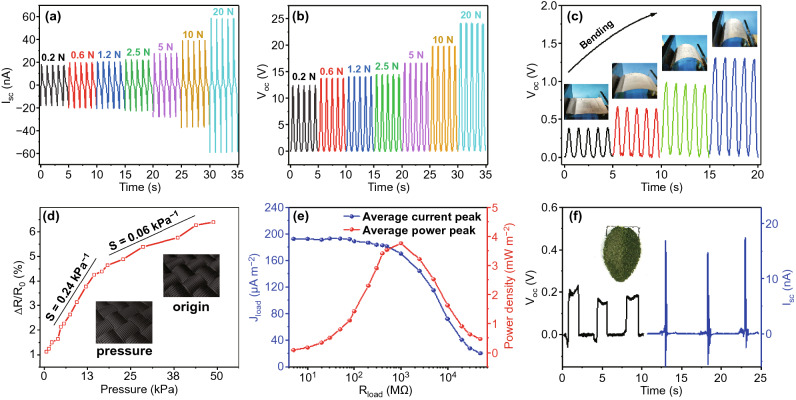
Pressure response of the F-TENG. **a-b** Electrical output of the F-TENG under different loading forces. **c** Effect of the degree of curvature on the *V*_oc_ of the F-TENG. The insets are the photographs of different bending states of the F-TENG. **d** Variation of resistance at different pressures. The insets are the schematic diagrams of the CNT/Cotton fabric under cycling loading and unloading pressure. **e** Instantaneous current density and power density varying with external resistance. **f** Optical image and electrical output of the F-TENG loading by a small leaf

In addition, the electrical output performance of F-TENG at two different linear changes with the increasing in pressure is shown in Fig. 3a, b and S5. The changes in electrical resistance caused by the elastic microporous shrink of CNT only work when a relatively small stress is applied to the F-TENG. However, the pattern will be changed when the stress on the F-TENG exceeds a certain critical value. Due to the flexible woven structure, the internal resistance of F-TENG will be altered with the elastic deformation of polyester fiber. As shown in Fig. 3c, the output performance of TENG correspondingly accelerates with the increase in curvature radius. It can be seen that F-TENG has a widely applications in pressure sensors and bending strain sensors. As shown in Fig. 3d, the sensitivity is described according to the two modes of F-TENG pressure change. ∆*R* is the relative change in resistance, and *R*_0_ is resistance without applied pressure. By gently placing a leaf on its surface, the results of detection pressure limit for F-TENG show that the F-TENG has a good sensitivity (Fig. S6) and a stable electrical output (Fig. 3f). Aiming to further simulate the power output characteristics of F-TENG when the load is actually used, the average power density of the F-TENG was measured. On the basis of the average power density formula (2) and the Ohm's law (3),

2 $$P = (I^{2} \times R)/A$$

3 $$I = \frac{U}{R}$$

where *A* is the effective contact area, *U* is the voltage of external load, *I* is the current of external load, *R* is the resistance of external load. The contact frequency was fixed at 1 Hz, and *I* begins to show a stable trend with the progressively increase in *R* and then decreases rapidly. The dependence of the optimal power density and current density on resistance is shown in Fig. 3e. All of the F-TENGs reach their peak values at the resistance of about 1 GΩ. Their peak power density is 3.8 mW m^−2^, and the average current density is 170 µA m^−2^ at this point.

### Energy Harvesting and Powering Electric Devices

**Fig. 4:**
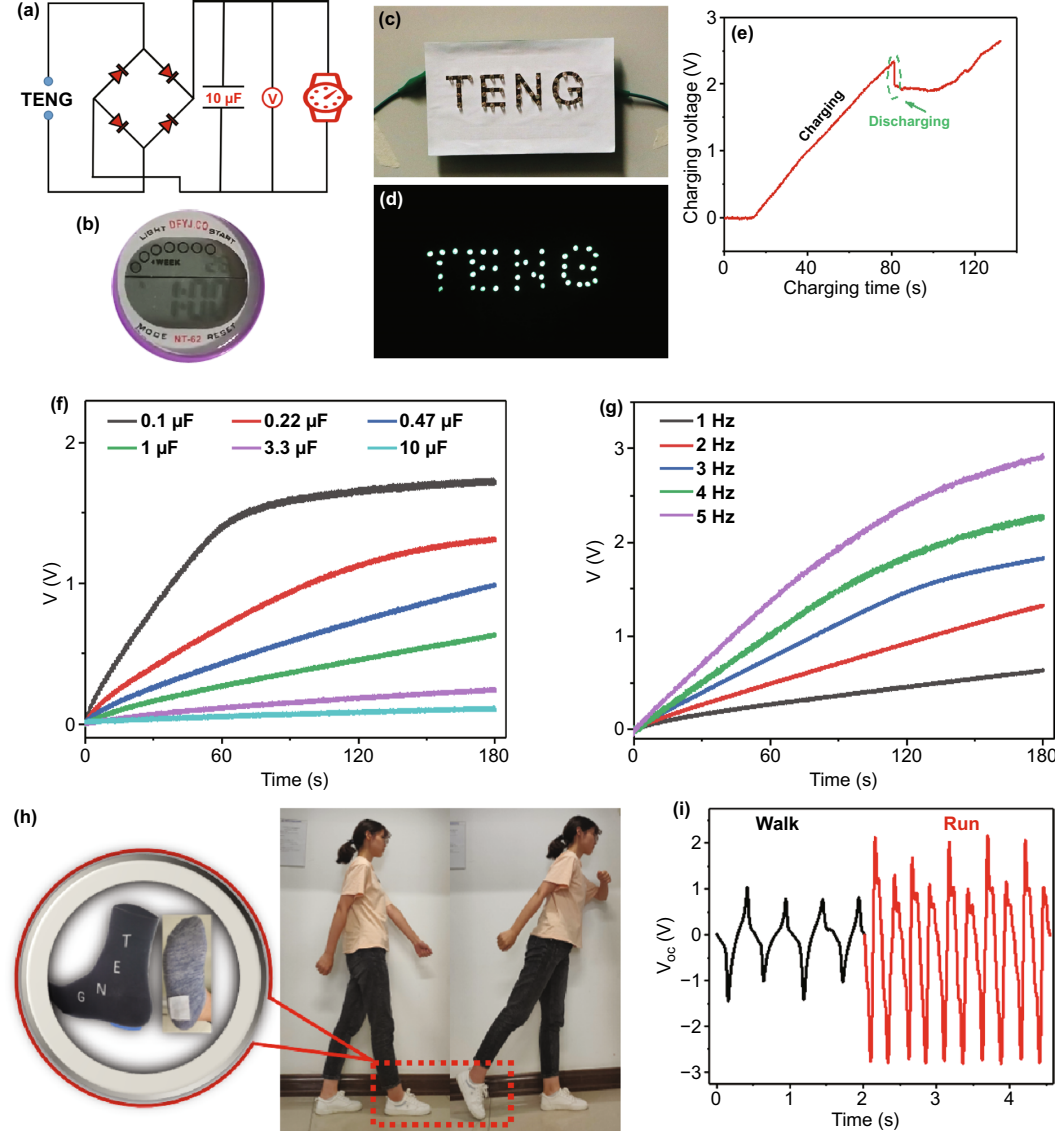
Energy-harvesting capability of the F-TENG. **a** Circuit diagram of the charging system for the F-TENG. **b** Photograph showing that a commercial watch is on the working state. **c**, **d** Demonstration of lighting up different LED units by tapping a single F-TENG. **e** Charging voltage of the F-TENG as a function of charging time. **f**, **g** Charging capability of the F-TENG under different capacitance capacities (1–10 µF) (**f**) and under different striking frequencies (1–5 Hz) (**g**). **h–i** A pedometer system fabricated by attaching the F-TENG on a sock. **h** Schematic diagram and **i** voltage signal output under the walking and running state

The F-TENG has excellent electrical output performance which can be realized by self-powered function. A commercial watch with a turn-on voltage of 1.5 V works normally when the F-TENG is continuously tapped at a normal frequency (Fig. 4a, b). The capacitor was used as an energy storage device to provide continuous power for the watch. In the charging phase of the initial stage, the frictional charges of F-TENG are continuously transferred to the 10 µF capacitor until it reaches the threshold voltage. Although the voltage of the capacitor drops off a cliff when the watch begins to work, it can still be charged up again through the continually striking (Fig. 4e). As an energy harvester, the F-TENG can light up 38 LEDs that are arranged into the letters "TENG" (Fig. 4c, d and Movie S1). This enables people to provide the energy required for lighting just by low-frequency swing or friction motion when walking at night. The differences in breakdown frequencies (1–5 Hz) and capacitance (0.1–10 µF) will have an important impact on the charging performance of F-TENG. The results indicate that the charging speed of F-TENG increases with a rise in tapping frequency, but the charging of the capacitor will slow down as the capacitor gradually reaches saturation (Fig. 4f, g).

Traditional pedometers are usually mechanized electronic products because of easy-to-lose and require external power. Based on the energy harvesting and high sensitivity functions, the F-TENG was designed as a wearable pedometer with the effective sensing area of 2 × 2 cm^2^ by sewing it on common socks (Fig. 4h). It is well known that the soles of human feet bear different ground pressures between the walking and running state, and the contact time is also different. Compared with the walking state, the F-TENG on the heel contacts more fully with the ground than that during the running state, bringing about a higher electrical output performance. Not only that, the difference of frequency that comes from the speed of putting foot down is also a well means to distinguish between running and walking state (Fig. 4i).

### Application of SPWK in Biometrics

**Fig. 5:**
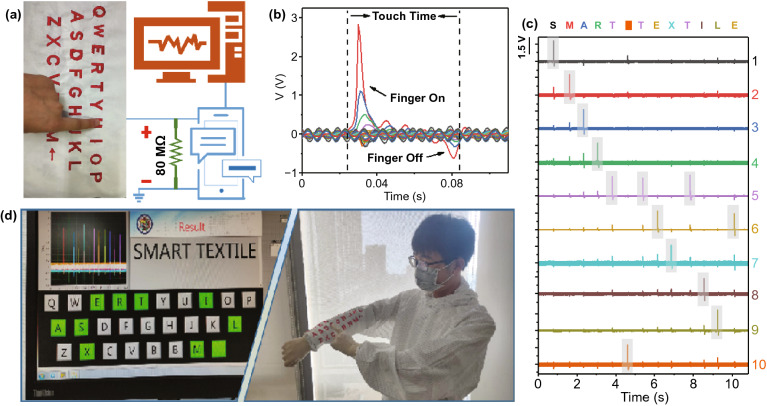
Working principle of the self-powered wearable keyboard as a biometric self-recognition system. **a** Schematic diagram of a SPWK working with a terminal device. **b** A diagram of a voltage signal when a finger strikes a single keypad. **c** Signal output waveform by continuously typing the string of "SMART TEXTILE" that is recorded in real time without uncomfortable delay. **d** Photograph showing the SPWK system for the real-time keystroke tracing and recording

With the advent of the information age, information security issues, such as the loss, leakage, and forgotten of digital passwords, are increasingly plaguing our lives. Keyboards, which can be used as the most commonly text input tool, are widely existing in human–computer interaction (Table S1), for instance, cash registers, automated banking machines, musical instrument, game machines [25, 45], etc. F-TENG owns multiple excellent characteristics of fabrics and TENGs. Therefore, it is a good candidate by combining F-TENG to form a large-area sensor array for constructing a SPWK. The working principle of the SPWK is shown in Fig. 5a, and the terminal device displays a digital signal which is converted from the electrical signal generated by pressing SPWK. Each electrical signal output of the measurement system is connected with a separate keyboard and acquisition card, and an 80 MΩ resistor is connected in parallel at the electrode channel to reduce the obstacles in environmental noise (Fig. S7). At the same time, a turn-on voltage of 2 V can be preset to ensure SPWK works stably even under high ambient noise.

When the keystroke on the particular position is initiated, a peak output voltage up to 2.8 V is acquired from the corresponding channel, while signals from other keys are less than 1.2 V (Fig. 5b). At this time, only the output voltage of buttons is higher than the threshold voltage 1.2 V. In theory, other channels cannot generate any electrical signals. However, the actual detection of weak electrical signals is derived from environmental noise and small changes in other channels. The keyboard would be working, while the positive charges of finger approach SPWK, and the voltage in the external circuit will continue until the operator's finger is completely separated from the top friction layer.

The threshold voltage is set to recognize and track the position of the touched keyboard. The Pauta criterion is used to analyze the corresponding critical voltage *V*_th_:

4 $$V_{{{\text{th}}}} = \frac{1}{n}\sum\limits_{i = 1}^{n} {V_{{{\text{pi}}}} + \frac{3}{\sqrt n }}$$

where *V*_pi_ is the peak voltage of the *i*_th_ channel, n is the number of all key channels, and *i* is the integer from 1 to n. Keystroke behavior is valid at the terminal device when the voltage signal was generated by the SPWK and was higher than the threshold value set. As shown in Fig. 5c, d the virtual keyboard on the PC side is tracked and located in real time when the corresponding keys of "SMART TEXTILE" are continuously typed. Each typematic behavior is accurately recorded without any delay (Movie S2 for a demonstration of keyboard inputting).

Since the electrical output generated by the SPWK is related to a variety of information, such as different subjects' slap speed, slap strength, finger size, and biological charge, a series of electrical signals produced by different individuals are dynamically recorded and analyzed. It can be seen from Fig. S8, the voltages are generated when different subjects press the same button, and significantly difference was occurred at three different subjects by repeat pressing four different keys more than 200 times. As we can see, for button "E", the average voltage produced by Tom per tap, Bob per tap, and Lee is 4.1, 2.9, and 1.3 V, respectively. Own to its good user identification, SPWK can accurately, uniquely, and persistently judge whether the user is a safe object. For this purpose, the electrical output signals of three different volunteers were collected when they pressed the same set of keys to analyze their respective characteristics.

**Fig. 6:**
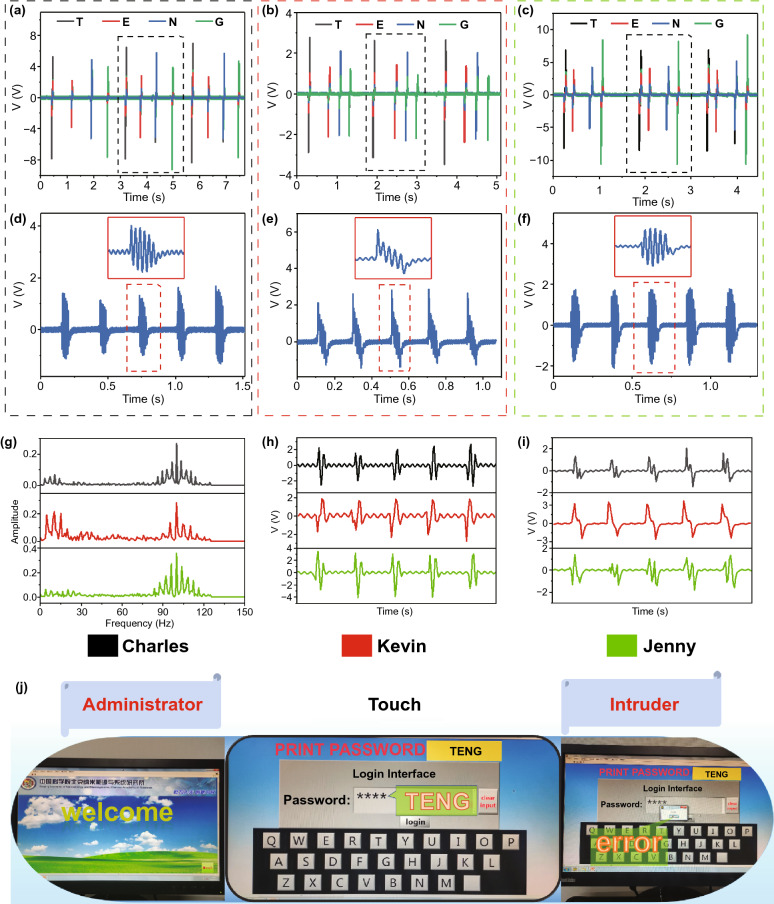
Application of the self-powered wearable keyboard as a biometric authentication. **a**–**c** Repeated voltage signals generated from typing the F-TENGs by the three volunteers. **d**–**f** Difference in voltage signal generated by different experimenters pressing the same button **a, d** Charles, **b, e** Kevin, and **c, f** Jenny. **g** Spectrogram through the Fourier transform from the signal of different objects. **h**, **i** Detailed waveform after the wavelet transform, ψ_3_ **h** and ψ_4_ **i**. **j** SPWK with self-security function used to identify administrators and intruders

The output voltage, tap interval, and signal waveform produced by different collection objects are different. According to the analysis of the tapping interval, the response time of subjects to the next keystroke is also inconsistent at the end of each keystroke, resulting in different characteristic frequencies for each group of strokes by different experimenters. (The figure is marked with a black dotted box, Fig. 6a-c.) The voltage signal of the same key "E" pressed by three subjects is used to further analyze the keystroke signal difference by different operating objects. As shown in Fig. 6d-f, on the one hand, the signals of Charles and Jenny show a longer response time by stay longer time at each press. On the other hand, the voltage signal of Kevin shows a larger amplitude because of its higher charge. All these features can be used as biometric judgments. As shown in Fig. S9, the average time by three experimenters stayed after pressing a word or a letter until the next pressing was recorded, the chart exhibits that the speed of each person's keyboard input is inconsistent with the reaction time after input.

The purpose of processing data is to calculate the frequency, amplitude, and phase of different sine wave signals by accumulating the complex original signal. The Fourier transform is used to transform the output signal from the time domain to the frequency domain in data processing. Three different characteristic signal waveforms are obtained, as shown in Fig. 6g. At the same time, the characteristic signals in the time domain and frequency domain can be captured from the discrete wavelet transformation (DWT). The detailed formula is as follows:

5 $$W_{j,k} (f) = \int\limits_{ - \infty }^{ + \infty } {\psi_{j,k} } (t)f(t){\text{d}}t$$

6 $$f\left( t \right) = \psi_{1} \left( t \right) + \psi_{2} \left( t \right) + \psi_{3} \left( t \right) + \psi_{4} \left( t \right)$$

Based on the Haar wavelet, the multi-resolution transformation of the original electrical signal is carried out to obtain the voltage components *ψ*_1_(*t*), *ψ*_2_(*t*), *ψ*_3_(*t*), and *ψ*_4_(*t*) at multiple scales (Figs. 6h-I and S10). *j* (*j* = 1, 2, 3, 4) is a positive integer. *k* is the number of changes in a given scale. *f*(t) is corresponding to the voltage signal in the original state, and *ψ*_2_, *ψ*_3_, and *ψ*_4_ are the detail component after the source voltage was decomposed. To achieve a highly secure biometric security management, a secure login system was constructed with LabVIEW by using the difference in electrical output signals between different operators. Firstly, we preset the administrator's threshold voltage, tap frequency, tap time, and other biological characteristics and then select three different users to enter the preset passwords. The result shows that even if the digital password is exposed, there is still only a specific matched user can successfully enter the system (Fig. 6j and Movie S3). It should be noted that signal analysis for calculating the characteristic value and setting the matching degree of the voltage signal can further improve the biometric recognition capability of the SPWK.

In addition, it is worth noting that the contact electrification can be appeared at any two materials, which means that the input response of SPWK is not only from fingers, but also from all other insulators and even conductive materials. Thus, the electrical performance of different gloves shows that the electrical output of the PE gloves is better than that of others (Fig. S11).

## Conclusions

In summary, a stretchable and conformable F-TENG as a wearable SPWK with feature of biological recognition is developed through a simple, low cost, and easily scalable approach. The self-powered fabric sensor is formed by silver-plated cloth combined with CNT- and PTFE-coated fabric. The fabricated F-TENG has desired stretchability, good sensitivity, high detection resolution, and fast response time. As an energy harvester, the F-TENG can produce an instantaneous average power current density up to 170 µA m^−2^, which is capable of lighting up 38 LEDs with the area of 2 × 2 cm^2^, charging capacitors sustainably, and powering an electronic watch, etc. The F-TENG is further sewed into a large-area pressure sensor array as a SPWK. The SPWK has biological recognition function, which can dynamically identify the operation users. Considering the exceptional properties of wearability, self-safety, self-power supply, low-cost, high-accuracy, and contact electrification performance, the SPWK has practical applications in human–computer interaction devices and personal user identification systems. The justified concepts and demonstrations in this work can be flexibly and extensively adopted in a variety of applications, ultimately improving our living way.

## Supplementary Information

Below is the link to the electronic supplementary material.

Supplementary file1 (MP4 1326 KB)

Supplementary file2 (MP4 8601 KB)

Supplementary file3 (MP4 4871 KB)

Supplementary file4 (DOCX 1456 KB)
