# Supplementary material for: Fully Fabric-Based Triboelectric Nanogenerators as Self-Powered Human–Machine Interactive Keyboards
Source: Nanomicro Lett. 2021 Apr 5;13:103. doi: 10.1007/s40820-021-00621-7 (PMC8021621; doi:10.1007/s40820-021-00621-7)
Supplement: Supplementary file 4 — Supplementary file4 (DOCX 1456 KB) [file 40820_2021_621_MOESM4_ESM.docx]

**Supporting Information**

**Fully Fabric-Based Triboelectric Nanogenerators as Self-Powered Human-Machine Interactive Keyboards**

Jia Yi ^1,2,#^, Kai Dong ^2,3,#^, Shen Shen ^2^, Yang Jiang ^2,3^, Xiao Peng ^2,3^ , Cuiying Ye ^2,3^, Zhong Lin Wang ^2,3,4,*^

^1^ School of Physical Science and Technology, Guangxi University, Nanning 530004, P. R. China

^2^ CAS Center for Excellence in Nanoscience, Beijing Key Laboratory of Micro-Nano Energy and Sensor, Beijing Institute of Nanoenergy and Nanosystems, Chinese Academy of Sciences, Beijing 100083, P. R. China

^3^ School of Nanoscience and Technology, University of Chinese Academy of Sciences, Beijing 100049, P. R. China

^4^ School of Material Science and Engineering, Georgia Institute of Technology, Atlanta, GA 30332-0245, USA

^#^ These authors contributed equally to this work.

^*^ Corresponding author: Zhong Lin Wang. E-mail: zhong.wang@mse.gatech.edu


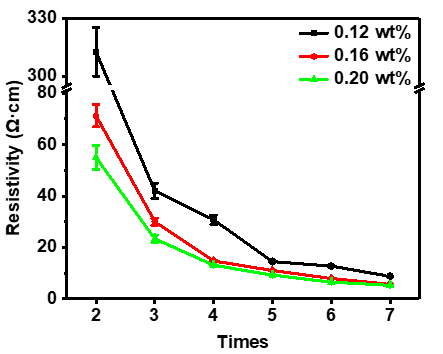


**Figure S1.** The resistivity of CNT/Cotton immersion with different concentrations of CNT solution varied with the number of immersion times.


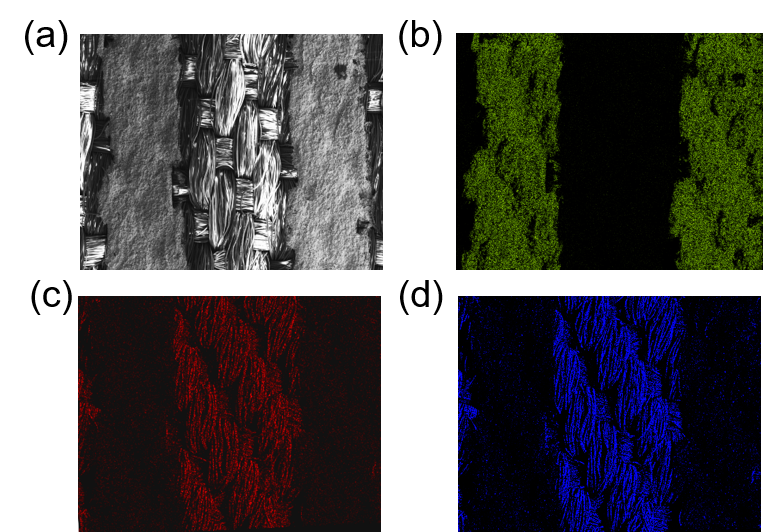


**Figure S2.** Elemental mapping (EDS) of the surface of the Silver-coated fabric. The elements C and O are from the polyester fabric. Ag microparticles are regularly embedded in the polyester textile.


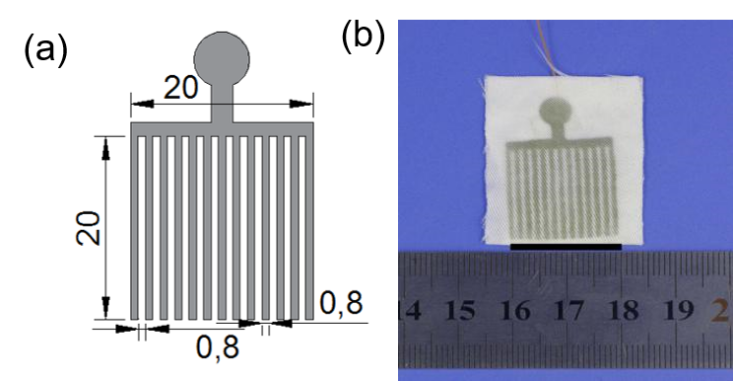


**Figure S3.** Dimension drawing and optical picture of the fork-shaped electrode. The coated silver paste is designed into a fork-shaped electrode pattern with a size of 2×2 cm2. The width of the forked electrode is 0.8 mm, and the distance between their insulation gaps is 0.8 mm


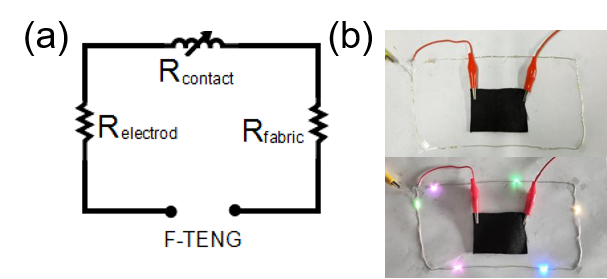


**Figure S4. (a)** Operating principle and circuit schematic of the F-TENG with and without applied pressure. **(b)** CNT fabric to be connected in series as conductor trace line in a circuit and maintain good connectivity. The resultant resistance of the device is the sum of three components: namely, the resistances of the bottom interdigitated electrode (R_electrode_), contact interface (R_contact_), and top CNT fabric film electrode (R_film_) as given in Equation (1):

$R_{total}\approx R_{electrode}+ R_{contact}+ R_{film}$ (1)

As the Ag electrodes employed in this work are highly conductive, Equation (1) can be reduced to

$R_{total}\approx R_{contact}+ R_{film}$ (2)

It is found that in general, and R_contact_ is more than an order higher than R_film_. While both R_contact_ and R_film_ reduce with increased pressure applied to the device, the variation in R_contact_ is much more significant than that in R_film_. The F-TENG response is dominated by R_contact_, i.e., the resistance formed between the rough contact area of CNT fabric and the Ag interdigitated textile.


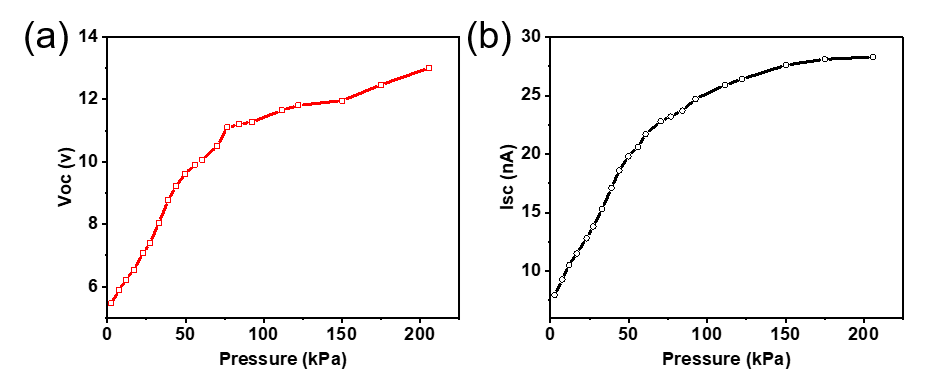


**Figure S5. a-b** Change curve of the VOC, ISC under different pressures.


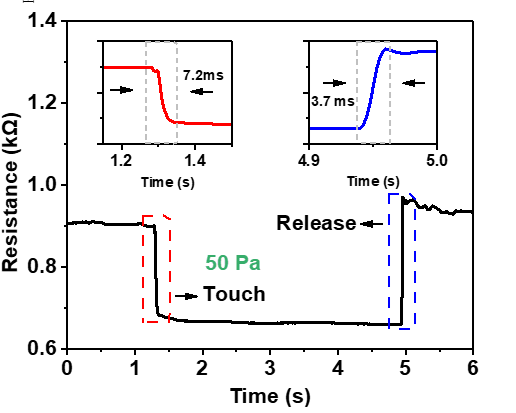


**Figure S6.** The response time of the F-TENG under pressure of 50 Pa.


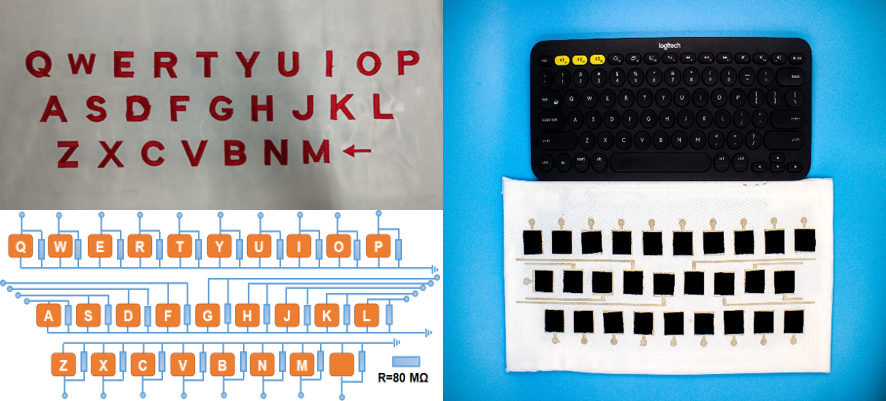


**Figure S7.** Optical image and underlying electrode circuit diagram of the SPWK at the left. A comparison of SPWK and a “Logitech” commercial keyboard.


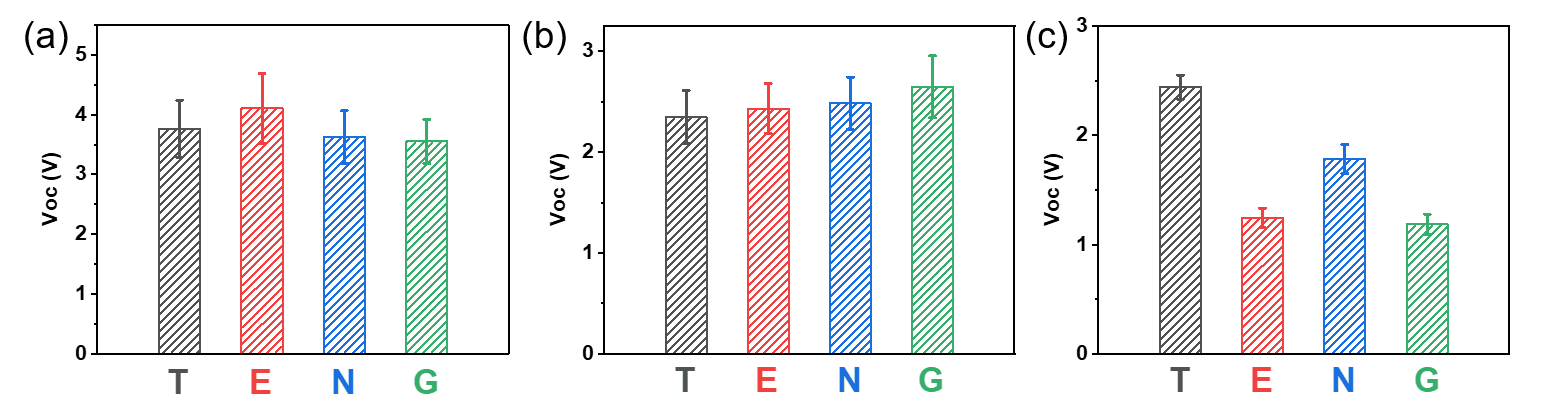


**Figure S8.** Error range of the peak voltage and peak current signals, which was obtained when (a) Charles, (b) Kevin, (c) Jenny, was continuously tying the word touch more than four times into the computer via the SPWK.


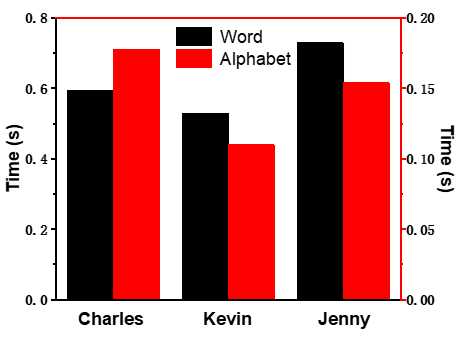


**Figure S9.** The reaction time required for Charles, Kevin and Jenny pressing word “TENG” and pressing alphabet “T”, “E”, “N”, “G”.


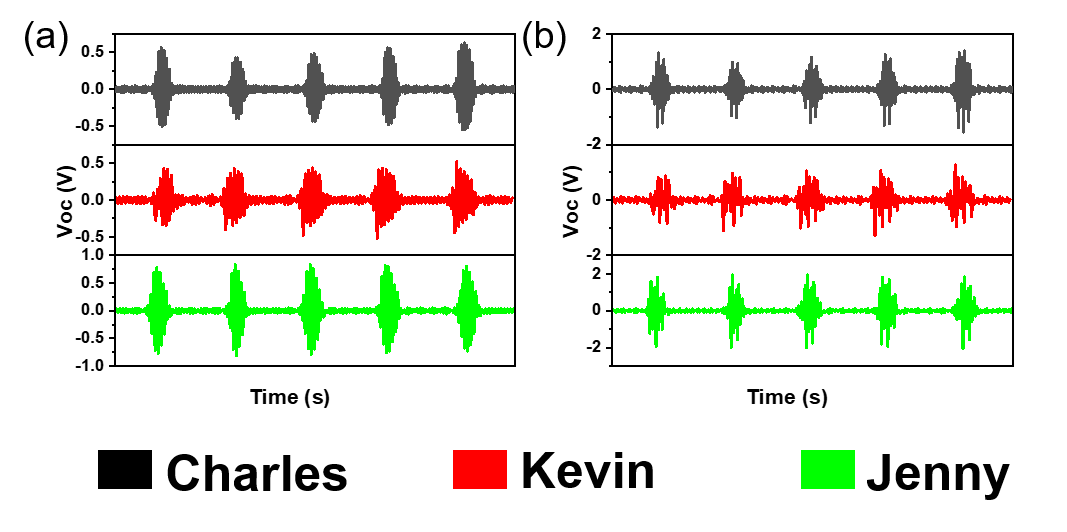


**Figure S10.** The higher resolution terms (a) ψ1 and (b) ψ2 of original typing patterns after DWT, respectively for Charles, Kevin and Jenny. As shown, these higher resolution wavelet components of the typing patterns are significantly different from each other.


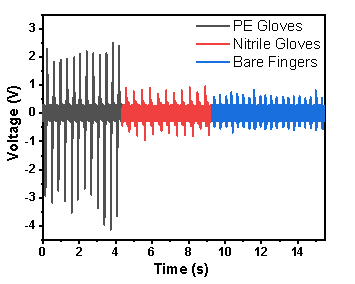

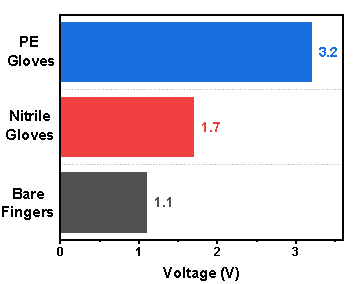


**Figure S11.** The signal output of sPE Gloves, Nitrile Gloves and Bare Fingers.

**Table S1.** The characteristics of different human-computer interaction devices

| Device | Wearable mode | Signal source | Application |
| --- | --- | --- | --- |
| Textile glove [1] | Sewing | Triboelectricity | Gesture recognition |
| Polymer glove [2] | Integration | Capacitance | Pressure measurement |
| Handwriting pad [3] | - | Triboelectricity | Biometric |
| Interacting patch [4] | Cover | Triboelectricity | Pressure measurement |
| Textile keyboard [5] | Sewing | Triboelectricity | Signal input |
| Polymer keyboard [6] | - | Triboelectricity | Biometric |
| Semiconductor patch [7] | Cover | Field Effect Transistor | Movement monitoring |
| Textile touchpad [8] | Integration | Triboelectricity | pressure distribution |
| Polymer microphone [9] | - | Soft Resistive | Voice recognition |
| Polymer braille screen [10] | - | Triboelectricity& Elastomer | Signal output |

**Reference**

1. B. Dong, Y. Yang, Q. Shi, S. Xu, Z. Sun, S. Zhu, Z. Zhang, D.-L. Kwong, G. Zhou, K.-W. Ang, C. Lee. Wearable triboelectric–human–machine interface (thmi) using robust nanophotonic readout. ACS Nano. **14**(7), 8915-8930 (2020). https://doi.org/10.1021/acsnano.0c03728

2. S. Zhao, W. Ran, D. Wang, R. Yin, Y. Yan, K. Jiang, Z. Lou, G. Shen. 3d dielectric layer enabled highly sensitive capacitive pressure sensors for wearable electronics. ACS Appl. Mater. Inter. **12**(28), 32023-32030 (2020). https://doi.org/10.1021/acsami.0c09893

3. W. Zhang, L. Deng, L. Yang, P. Yang, D. Diao, P. Wang, Z. L. Wang. Multilanguage-handwriting self-powered recognition based on triboelectric nanogenerator enabled machine learning. Nano Energy. **77,** (2020). https://doi.org/10.1016/j.nanoen.2020.105174

4. G. Tang, Q. Shi, Z. Zhang, T. He, Z. Sun, C. Lee. Hybridized wearable patch as a multi-parameter and multi-functional human-machine interface. Nano Energy. **81,** (2021). https://doi.org/10.1016/j.nanoen.2020.105582

5. S.-B. Jeon, S.-J. Park, W.-G. Kim, I.-W. Tcho, I.-K. Jin, J.-K. Han, D. Kim, Y.-K. Choi. Self-powered wearable keyboard with fabric based triboelectric nanogenerator. Nano Energy. **53,** 596-603 (2018). https://doi.org/10.1016/j.nanoen.2018.09.024

6. J. Chen, G. Zhu, J. Yang, Q. S. Jing, P. Bai, W. Q. Yang, X. W. Qi, Y. J. Su, Z. L. Wang. Personalized keystroke dynamics for self-powered human-machine interfacing. ACS Nano. **9**(1), 105-116 (2015).

7. Z. R. Kyoseung Sim, Zhanan Zou, Faheem Ershad, Jianming Lei, Anish Thukral, Jie Chen, Qing-An Huang, Jianliang Xiao, Cunjiang Yu. Metal oxide semiconductor nanomembrane–based soft unnoticeable multifunctional electronics for wearable human-machine interfaces. Sci. Adv. **5**(8), (2019). https://doi.org/10.1126/sciadv.aav9653

8. C. Ning, K. Dong, R. Cheng, J. Yi, C. Ye, X. Peng, F. Sheng, Y. Jiang, Z. L. Wang. Flexible and stretchable fiber‐shaped triboelectric nanogenerators for biomechanical monitoring and human‐interactive sensing. Adv. Funct. Mater. (2020). https://doi.org/10.1002/adfm.202006679

9. S. Gong, L. W. Yap, Y. Zhu, B. Zhu, Y. Wang, Y. Ling, Y. Zhao, T. An, Y. Lu, W. Cheng. A soft resistive acoustic sensor based on suspended standing nanowire membranes with point crack design. Adv. Funct. Mater. **30**(25), (2020). https://doi.org/10.1002/adfm.201910717

10. X. Qu, X. Ma, B. Shi, H. Li, L. Zheng, C. Wang, Z. Liu, Y. Fan, X. Chen, Z. Li, Z. L. Wang. Refreshable braille display system based on triboelectric nanogenerator and dielectric elastomer. Adv. Funct. Mater. (2020). https://doi.org/10.1002/adfm.202006612
